# Determinants and Mechanisms of the Low Fusogenicity and High Dependence on Endosomal Entry of Omicron Subvariants

**DOI:** 10.1128/mbio.03176-22

**Published:** 2023-01-10

**Authors:** Panke Qu, John P. Evans, Chaitanya Kurhade, Cong Zeng, Yi-Min Zheng, Kai Xu, Pei-Yong Shi, Xuping Xie, Shan-Lu Liu

**Affiliations:** a Center for Retrovirus Research, The Ohio State University, Columbus, Ohio, USA; b Department of Veterinary Biosciences, The Ohio State University, Columbus, Ohio, USA; c Molecular, Cellular, and Developmental Biology Program, The Ohio State University, Columbus, Ohio, USA; d Department of Biochemistry and Molecular Biology, University of Texas Medical Branch, Galveston, Texas, USA; e Viruses and Emerging Pathogens Program, Infectious Diseases Institute, The Ohio State University, Columbus, Ohio, USA; f Department of Microbial Infection and Immunity, The Ohio State University, Columbus, Ohio, USA; Indiana University Bloomington

**Keywords:** Omicron subvariants, furin cleavage, fusogenicity, endosomal entry, H655Y

## Abstract

The rapid spread and strong immune evasion of the SARS-CoV-2 Omicron subvariants has raised serious concerns for the global COVID-19 pandemic. These new variants exhibit generally reduced fusogenicity and increased endosomal entry pathway utilization compared to the ancestral D614G variant, the underlying mechanisms of which remain elusive. Here, we show that the C-terminal S1 mutations of the BA.1.1 subvariant, H655Y and T547K, critically govern the low fusogenicity of Omicron. Notably, H655Y also dictates the enhanced endosome entry pathway utilization. Mechanistically, T547K and H655Y likely stabilize the spike trimer conformation as suggested by increased molecular interactions in structural modeling and enhanced S1 shedding of their reversion mutants K547T and Y655H in viral producer cells. Importantly, the H655Y mutation also determines the low fusogenicity and enhanced dependence on the endosomal entry pathway of other Omicron subvariants, including BA.2, BA.2.12.1, BA.4/5, and BA.2.75. Together, these results uncover mechanisms governing Omicron subvariant entry and provide insights into altered Omicron tissue tropism and pathogenesis.

## INTRODUCTION

The emergence and rapid spread of the Omicron subvariants of SARS-CoV-2 around the world has caused serious concern about vaccine efficacy because of the large numbers of mutations present in this lineage, with more than 30 substitutions in the spike (S) proteins (1). Indeed, many recent studies have shown that Omicron is resistant to neutralization by antibodies induced by two-dose mRNA vaccination that is greatly restored by booster vaccination (2–8). Consequently, Omicron led to a global surge of COVID-19 cases. BA.1.1 and BA.1 were responsible for the initial Omicron wave but were replaced by the BA.2 subvariant, which was more transmissible and caused reinfection in patients who previously were infected by BA.1 (9). Remarkably, derivatives of BA.2, including BA.2.12.1, which subsequently became predominant in the United States, and the BA.4 and BA.5 subvariants bearing identical S proteins (referred to as BA.4/5 hereafter), which are currently dominant in the world, are driving a new surge in cases, as both have stronger immune escape ability, especially the BA.4/5 variants (10–13). Despite the gradually improved virus transmissibility observed in these subvariants, they appear to cause milder disease than Delta and other previous variants (14, 15). The mechanism underlying the reduced pathogenicity of Omicron is currently unclear but has been under intense investigation.

The SARS-CoV-2 S protein is a typical class I viral fusion protein that utilizes the cognate receptor angiotensin-converting enzyme 2 (ACE2) for binding and cellular entry (16, 17). It has a furin cleavage site at the S1/S2 junction of S, which facilitates SARS-CoV-2 entry at the plasma membrane, viral replication in human lung epithelial cells, and transmission in animals (18, 19). As SARS-CoV-2 evolves, its S has gained a large number of mutations and possibly undergone recombination, resulting in the emergence of the initial D614G variant and several major variants of concern (VOCs) such as Alpha, Beta, Gamma, Delta, and Omicron, some of which directly or indirectly increase S cleavage to enhance fusogenicity (20–22). Intriguingly, results from many groups, including ours, have shown that the Omicron subvariants exhibit substantially impaired cell-cell fusion capacity and tend to use the endosomal entry pathway mediated by cathepsin L/B (Cat L/B), rather than the plasma membrane entry pathway mediated by transmembrane protease serine 2 (TMPRSS2), which is preferred by previous variants (23–25).

Currently, the underlying molecular mechanism by which Omicron subvariants use endosomal entry more efficiently than plasma membrane entry is unclear. Here, we provide evidence that some key mutations in S1 and/or the S1/S2 junction region of BA.1.1 S, i.e., T547K and H655Y, especially the latter, dictate its intrinsically low fusogenicity and endosomal entry. Mechanically, we find that T547K and H655Y restrict BA.1.1 S-mediated cell-cell fusion, possibly through stabilizing the S trimer conformation. H655Y, but not T547K, which is only carried by BA.1.1 and BA.1 of Omicron subvariants (13), governs the entry preference and fusion capability of all predominant Omicron subvariants. Together, our results reveal that mutations in S1 around the furin cleavage site of Omicron S critically modulate the unique biology of Omicron entry and are potentially associated with pathogenesis.

## RESULTS

### Critical amino acid residues dictating the differential entry patterns of Omicron subvariant BA.1.1 in HEK293T-ACE2 and Calu-3 cells.

**FIG 1:**
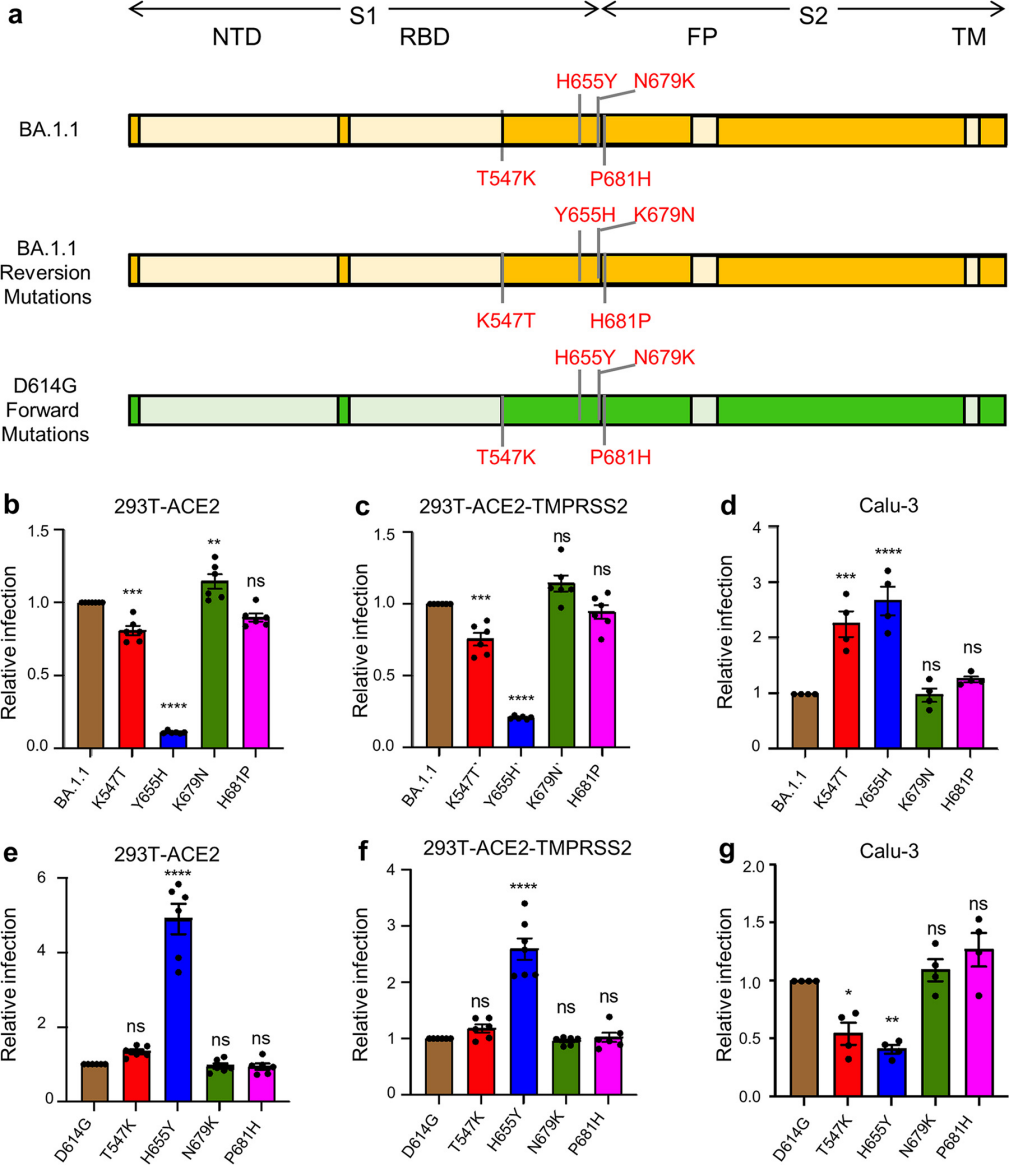
H655Y governs differential entry of Omicron BA.1.1 into distinct target cells. (a) Schematic representations of BA.1.1 and D614G spike glycoprotein are presented. The N-terminal domain (NTD), the receptor binding domain (RBD), the fusion peptide (FP), and the transmembrane (TM) region are indicated. Only the mutations at the C terminus of S1 and those near the S1/S2 junction in BA.1.1 S relative to SARS-CoV-2 D614G S are shown (top, BA.1.1) (43); also displayed are these four amino acid mutations of BA.1.1 S replaced with the corresponding amino acid in the D614G S (middle, BA.1.1 reversion mutations) and these four residues in D614G S substituted with the corresponding amino acid in BA.1.1 S (bottom, D614G forward mutations). (b to d) The relative infectivity of pseudotyped viruses encoding BA.1.1 S with single mutated amino acids replaced with the corresponding amino acid in the D614G S in HEK293T-ACE2 cells (*n* = 6) (b), HEK293T-ACE2-TMPRSS2 cells (*n* = 6) (c), and Calu-3 cells (*n* = 4) (d). The luciferase activity of parental BA.1.1 was set as 1.0 for comparison. (e to g) The relative infectivity of pseudotyped viruses encoding D614G S with single amino acids substituted with the corresponding amino acid in the BA.1.1 S in HEK293T-ACE2 cells (*n* = 6) (e), HEK293T-ACE2-TMPRSS2 cells (*n* = 6) (f), and Calu-3 cells (*n* = 4) (g). The luciferase activity of parental D614G was set as 1.0. In all cases, bars represent means ± standard error, and significance was determined by one-way ANOVA with Bonferroni’s multiple testing correction. ns, *P* ≥ 0.05; *, *P* < 0.05; **, *P* < 0.01; ***, *P* < 0.001; ****, *P* < 0.0001.

Previous studies have shown that SARS-CoV-2 entry is cell-type dependent, depending on the levels of TMPRSS2 expression on the plasma membrane of target cells (17, 26). In low-TMPRSS2-expressing 293T-ACE2 and Vero cells, the endosomal entry pathway is predominant; however, in Calu-3 and other cells expressing high levels of TMPRRS2, SARS-CoV-2 primarily uses the plasma membrane for entry (17). Of note, stable expression of TMPRSS2 in 293T-ACE2-TMPRSS2 cells allows for efficient usage of both entry pathways (23). In addition, entry of SARS-CoV-2 is modulated by the efficiency of furin cleavage of the virus S protein; for example, deletion or mutation of the furin cleavage site can substantially impair this viral entry even in cells with high levels of TMPRSS2 (18). Given that D614G mutation is not in the furin cleavage site but can dramatically impact SARS-CoV-2 S furin cleavage and entry efficiency (27), we hypothesized that mutations around D614G, or near the furin cleavage site of Omicron S, potentially govern its endosomal entry. To test this hypothesis, we created reversion mutations specific to residues T547K, H655Y, N679K, and P681H of the Omicron subvariant BA.1.1 (Fig. 1a) and examined their impact on the entry of BA.1.1 into HEK293T-ACE2, HEK293T-ACE2-TMPRSS2, and Calu-3 cells using our previously reported HIV lentiviral pseudotyping system (28). In parallel, the entry in these cell types was tested for the corresponding forward mutations, i.e., T547K, H655Y, N679K, and P681H made in the backbone of the ancestral D614G construct (Fig. 1a). We found that, compared with parental BA.1.1, the reversion mutation Y655H exhibited a profound reduction of entry efficiency in HEK293T-ACE2 (Fig. 1b), and in HEK93T-ACE2-TMPRSS2 cells, albeit to a lesser extent (Fig. 1c), yet apparently enhanced entry in Calu-3 cells (Fig. 1d), suggesting that H655Y is the most critical change in BA.1.1 S that distinguishes its entry in these different cell types. Consistent with this, we observed that the forward mutation H655Y exhibited reduced entry efficiency in Calu-3 cells but increased entry in 293T-ACE2 and 293T-ACE2-TMPRRS2 cells (Fig. 1e to g). While similar results were also observed for the BA.1.1 K547T mutation, the impact on entry was not as dramatic as that of H655Y (Fig. 1b to g). Surprisingly, two other mutations of BA.1.1, N679K and P681H, which are in close proximity to the furin cleavage site, did not seem to obviously affect viral entry in these cell types (Fig. 1b to g).

### H655Y governs the endosomal entry pathway of BA.1.1 and BA.1: differential effects of E64d and camostat.

**FIG 2:**
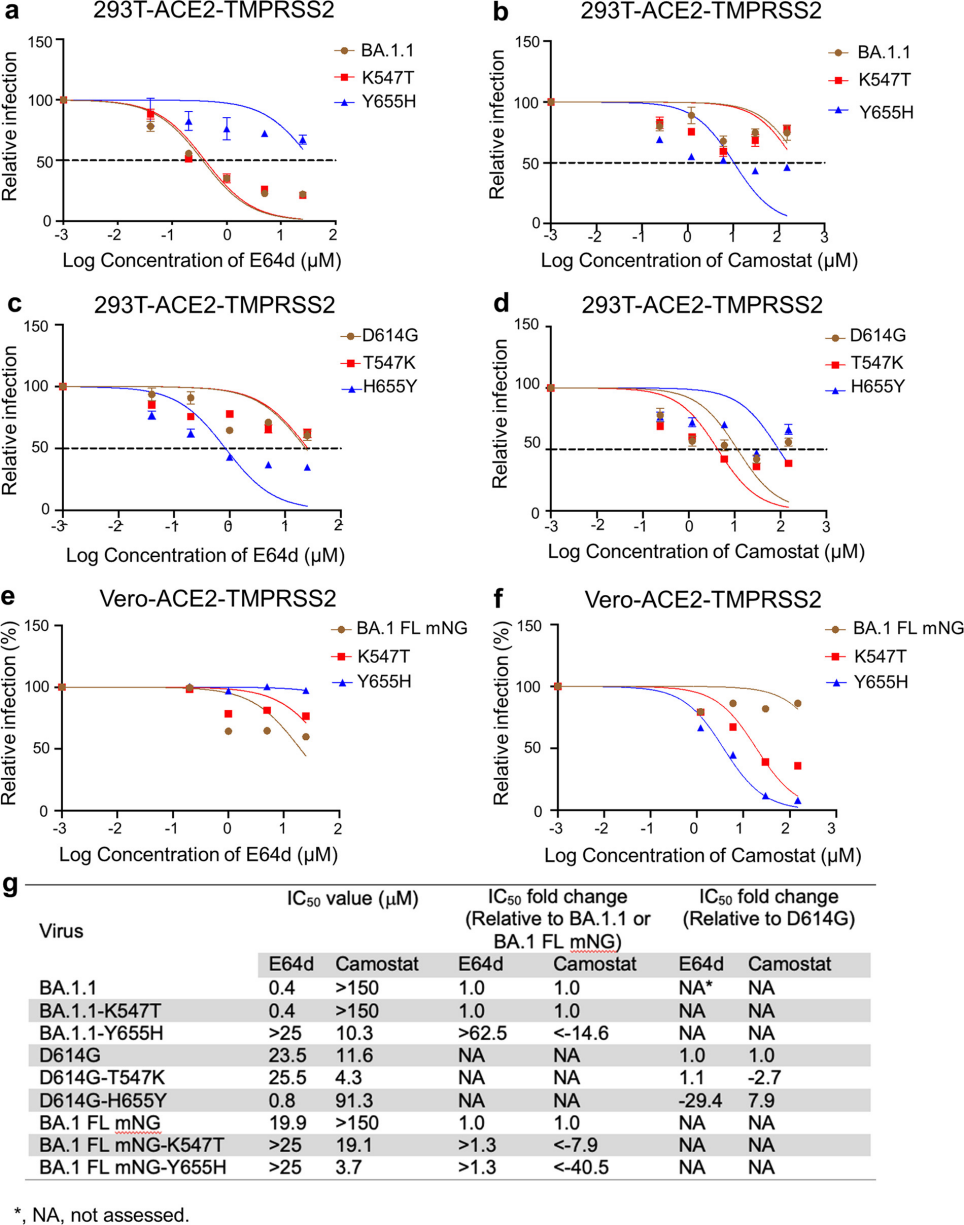
H655Y dictates BA.1.1 entry through the endosomal pathway. (a to d) HEK293T-ACE2-TMPRSS2 cells were pretreated with E64d (a and c) or Camostat (b and d) and then transduced with the indicated pseudotyped viruses in the presence of various concentrations of the inhibitors. The relative infection was calculated by setting the luciferase activity of each virus at the 0 μM the inhibitors as 100%, and the half maximal inhibitory concentration (IC_50_) values were determined by nonlinear regression with least-squares fit. (e and f) Vero-ACE2-TMPRSS2 cells were pretreated with E64d (e) or Camostat (f), followed by infection with parental BA.1 FL mNG or its mutants. The infection ratio was determined by flow cytometry, and relative infection was calculated by setting the infection percentage of each virus in the absence of the drugs as 100%. (g) IC_50_ values of each virus in the presence of the individual drugs are shown. Additionally, the IC_50_ fold changes relative to parental BA.1 (or BA.1 FL mNG) or D614G are displayed.

Previous studies have shown that SARS-CoV-2 entry in HEK293T-ACE2 cells is predominantly endosomal, whereas in Calu-3 cells entry is primarily through the plasma membrane (19). We thus decided to use HEK293T-ACE2-TMPRSS2 cells, which allow entry through both endosomal and plasma membrane routes, to determine the impact of these BA.1.1 mutants on entry in the presence of the endosomal Cat L/B inhibitor E64d or the TMPRSS2 inhibitor camostat mesylate (camostat). While BA.1.1 was more sensitive to treatment by E64d but less sensitive to treatment by camostat than D614G, BA.1.1-Y655H was less sensitive to E64d than BA.1.1, with a half maximal inhibitory concentration (IC_50_) of > 25 μM versus an IC_50_ of 0.4 μM for the parental BA.1.1 (Fig. 2a and g). Additionally, BA.1.1-Y655H was more sensitive to camostat than BA.1.1, with an IC_50_ of 10.3 μM versus an IC_50_ of >150 μM for BA.1.1 (Fig. 2b and g). Convincingly, the forward mutant D614G-H655Y was more sensitive to E64d, with an IC_50_ of 0.8 μM versus 23.5 μM for the parental D614G (Fig. 2c and g), but less sensitive to camostat, with an IC_50_ of 91.3 μM versus 11.6 μM for the parental D614G (Fig. 2d and g). Overall, we discovered that the BA.1.1 reversion mutant Y655H had a more than 14.6-fold-decreased IC_50_ for camostat and an over 62.5-fold-increased IC_50_ for E64d compared to the parental BA.1.1 (Fig. 2g). Further, the forward mutant H655Y has a 7.9-fold-increased IC_50_ for camostat and a 29.4-fold-decreased IC_50_ for E64d relative to the parental D614G (Fig. 2g). Of note, the T547K mutation of BA.1.1 did not seem to have a dramatic impact on the sensitivity to these drug treatments (Fig. 2a to d and g).

To confirm the above-described pseudotype lentivirus results in an authentic SARS-CoV-2 system, we engineered the complete BA.1 (BA.1 contains the same mutations as BA.1.1 except for the R346K mutation, which is absent in BA.1) full-length (FL) spike (4, 29) into the infectious cDNA clone of USA-WA1/2020 with mNeonGreen (1) reporter (30) to obtain BA.1-FL mNG SARS-CoV-2 and then generated the individual reversion mutations of spike K547T or Y655H in this background. Using these infectious viruses, we determined the impact of these two mutations on BA.1 entry into Vero-ACE2-TMPRSS2 cells using the inhibitor E64d or camostat. The results showed that the parental BA.1-FL mNG SARS-CoV-2 was the most sensitive to E64d, with an IC_50_ of 19.9 μM, among the three viruses, followed by BA.1-FL mNG-K547T and then BA.1-FL mNG-Y655H, although these variants were generally much less sensitive to E64d than that in 293T-ACE2 cells, with no exact IC_50_ value calculated for the two mutants (Fig. 2e and g; see Fig. S1a in the supplemental material), possibly due to the different cell lines used, the experimental procedures, and/or the R346K mutation, which is absent in BA.1. In contrast, compared to the parental BA.1-FL mNG, which was resistant to camostat treatment with an IC_50_ of more than 150 μM, BA.1-FL mNG-K547T was sensitive to camostat with an IC_50_ of 19.1 μM, a more than 7.9-fold reduction in IC_50_ relative to BA.1-FL mNG; BA.1-FL mNG-Y655H was more sensitive to camostat, with an IC_50_ of 3.7 μM, an approximately >40.5-fold-decreased IC_50_ relative to BA.1 FL mNG (Fig. 2f and g; Fig. S1b). These results together indicated that T547K and H655Y mutations, especially the latter, greatly contribute to the altered entry preference of BA.1 in target cells. Overall, the results of pseudotyped as well as authentic viruses highlight the essential role of H655Y mutation in dictating the Omicron BA.1.1 or BA.1 subvariant S-mediated endosomal entry compared to the ancestral D614G, which enters target cells predominantly through the plasma membrane.

10.1128/mbio.03176-22.1 FIG S1 H655Y dictates BA.1.1 infection through the endosomal pathway. (a and b) Vero-ACE2-TMPRSS2 cells were pretreated with the indicated concentrations of E64d (a) or camostat (b) and then infected by the infectious SARS-CoV-2 viruses expressing mNeonGreen (1). After fixation, the cells were analyzed by flow cytometry. Representative flow cytometry plots showing the percent of infection are presented. Download FIG S1, TIF file, 2.5 MB. Copyright © 2023 Qu et al. 2023 Qu et al. https://creativecommons.org/licenses/by/4.0/ This content is distributed under the terms of the Creative Commons Attribution 4.0 International license.

### H655Y critically controls the low fusogenicity of BA.1.1 spike.

**FIG 3:**
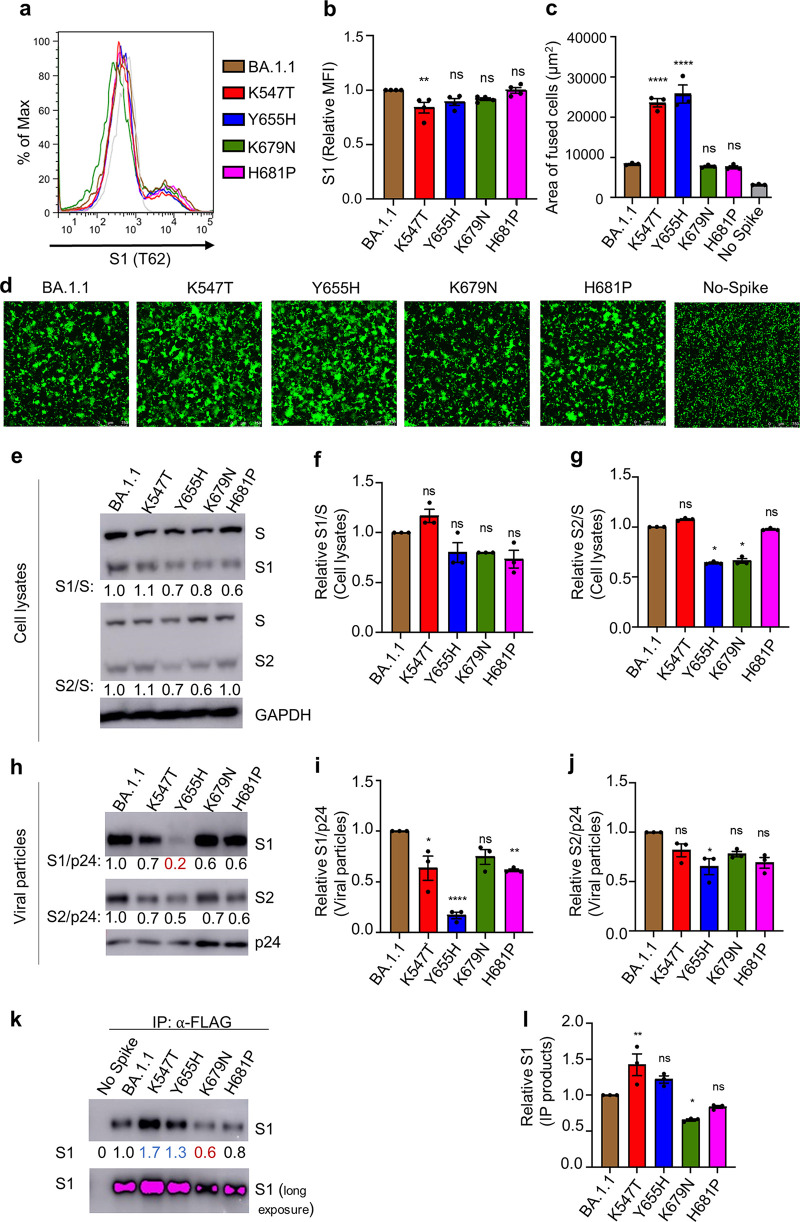
H655Y critically controls the low fusogenicity of BA.1.1 S. (a) The S expression on the cell surface of HEK293T cells used to produce pseudotyped lentivirus was determined by staining cells with anti-SARS-CoV-2 S1 (T62) antibodies. Representative flow cytometric histograms of the S1 signal are shown for the indicated S proteins. (b) Mean fluorescence intensity of S1 signal was calculated and shown (*n* = 4). (c and d) Syncytia formation was assayed in HEK293T-ACE2 cells transfected to express the S construct of interest and GFP. The average area of fused cells from one of five independent experiments (c) and representative syncytium images (d) are shown. Scale bars represent 150 μm. (e) Producer cell lysates were probed for S1 subunit, S2 subunit, HIV-1 p24, and GAPDH. S signals were quantified using NIH ImageJ, and the relative efficiency of S cleavage was calculated by setting the ratio of S1/S or S2/S of BA.1.1 to 1.00. Representative blots are shown. (f and g) The relative ratios of S1/S (f) and S2/S (g) from three independent experiments are shown. (h) The pseudovirions were purified and blotted for S1, S2, and HIV-1 p24, and S1 or S2 signals in virions were quantified and normalized to HIV-1 p24. (i and j) The relative S1/p24 or S2/p24 was calculated by setting the ratio of BA.1.1 to 1.00. The relative S1/p24 (i) and the relative S2/p24 (j) ratios from three independent experiments are shown. (k and l) Reversion of H655Y to Y655H enhances BA.1.1 S1 shedding. HEK293T cells transfected with the indicated S constructs were treated with soluble ACE2-Fc (sACE2), followed by immunoprecipitation (IP) of S1 in the culture media by anti-FLAG beads. S in the IP products (k) was probed for SARS-CoV-2 S1, with GAPDH as a loading control. Relative S1 in the IP products is shown (l), calculated by setting the S1 signals of BA.1.1 to 1.0. The error bars in panels b, c, f, g, i, j, and l) are means ± standard error. ns, *P* ≥ 0.05; *, *P* < 0.05; **, *P* < 0.01; ****, *P* < 0.0001.

To investigate the underlying mechanism of the T547K and H655Y mutations in modulating BA.1.1 entry preference, we examined the role of these two mutants along with N679K, P681H, and parental BA.1.1 in S expression and S-mediated membrane fusion. In parallel, the impacts of the corresponding forward mutants were also investigated. Flow cytometric analysis of the S surface expression in HEK293T cells used to produce pseudotyped lentivirus showed that all these reversion mutants had approximately comparable levels of expression to each other and the parental BA.1.1, except for the reversion mutant K547T, which had modestly reduced surface expression (Fig. 3a and b), while all the forward mutants had similar levels of expression to D614G (Fig. S2a and b). We then conducted syncytia formation assays in HEK293T-ACE2 cells transfected to express green fluorescent protein (GFP) and the individual S constructs and observed that K547T and Y655H substantially promoted the S-mediated cell-cell fusion, whereas K679N and H681P had no effect (Fig. 3c and d). Surprisingly, while forward mutations H655Y and T547K slightly reduced the D614G S-induced syncytia, two other mutations, N679K and P681H, enhanced D614G S-mediated fusion (Fig. S2c and d), similar to some previous reports (31).

10.1128/mbio.03176-22.2 FIG S2 H655Y mutation moderately impairs D614G S-mediated syncytium formation. (a and b) Viral producer HEK293T cells were digested with EDTA/PBS and stained with anti-S1 (T62) antibody for flow cytometry. Representative flow cytometric analyses are shown in panel a, and mean fluorescence intensity (MFI) is shown in panel b (*n* = 4). (c and d) HEK293T-ACE2 cells were transfected with the indicated S plasmids and a GFP expression plasmid, followed by fluorescence imaging and quantification of syncytium formation. (c and d) Representative quantification (c) and images (d) are shown (*n* = 3). Scale bars represent 150 μm. Error bars represent means ± standard error. Significance was determined by one-way ANOVA with Bonferroni’s multiple testing correction. ns, *P* ≥ 0.05; **, *P* < 0.01; ***, *P* < 0.001. Download FIG S2, TIF file, 2.5 MB. Copyright © 2023 Qu et al. 2023 Qu et al. https://creativecommons.org/licenses/by/4.0/ This content is distributed under the terms of the Creative Commons Attribution 4.0 International license.

### Reversion of H655Y to Y655H results in increased BA.1.1 S1 shedding.

The efficiency of SARS-CoV-2 S cleavage by furin is known to be associated with membrane fusogenicity (21, 32). We next examined how these mutations might influence BA.1.1 S processing by analyzing the ratio of S1/S and S2/S in viral producer cells and purified viral particles. In cell lysates, while K547T exhibited a slightly increased or comparable ratio of S1/S and S2/S, H681P exhibited a decreased S1/S ratio but not S2/S ratio; Y655H and K679N showed a modestly decreased ratio of both S1/S and S2/S (Fig. 3e to g). However, none of the four forward mutants showed an obvious change in S1/S1 or S2/S ratios in the cell lysates, except for N679K, which exhibited a modestly increased S2/S ratio (Fig. S3a to c). Interestingly, we found that all four reversion mutants, most notably Y655H, showed a decreased level of S1 and S2 compared to the parental BA.1.1 in purified viral particles after being normalized by HIV-1 p24; p24 was used to measure the amount of pseudovirions purified, thus serving as a virus input control (Fig. 3h to j). Additionally, the corresponding forward mutations T547K and H655Y, especially the latter, substantially increased the S1/p24 and S2/p24 ratios in viral particles relative to D614G (Fig. S3d to f). The drastically decreased level of S1 for Y655H in viral particles corresponded to its significantly reduced infectivity in HEK293T-ACE2 and HEK293T-ACE2-TMPRSS2 cells, potentially due to premature inactivation by high-level expression of ACE2 in these cells, despite apparently increased infectivity in Calu-3 cells (Fig. 1b to d). These results, along with the obviously increased fusogenicity of Y655H, suggest that the H655Y mutation in BA.1.1 critically governs its low fusion activity and differential entry pathways between HEK293T-ACE2 and Calu-3 cells.

10.1128/mbio.03176-22.3 FIG S3 Mutation H655Y contributes to more S incorporation into BA.1.1 virions. (a and d)Viral producer cells and supernatant were collected and blotted for S1, S2, GAPDH, and HIV-1 p24 in cell lysates (a) and viral particles (d). (b, c, e, and f) The relative S1/S ratio in cell lysates (b), relative S2/S ratio in cell lysates (c), relative S1/p24 ratio in viral particles (e), and relative S2/p24 ratio in viral particles (f) are shown (*n* = 3). Error bars are means ± standard error. Significance was determined by one-way ANOVA with Bonferroni’s multiple testing correction. ns, *P* ≥ 0.05; *, *P* < 0.05; ***, *P* < 0.001; ****, *P* < 0.0001. Download FIG S3, TIF file, 2.5 MB. Copyright © 2023 Qu et al. 2023 Qu et al. https://creativecommons.org/licenses/by/4.0/ This content is distributed under the terms of the Creative Commons Attribution 4.0 International license.

**FIG 4:**
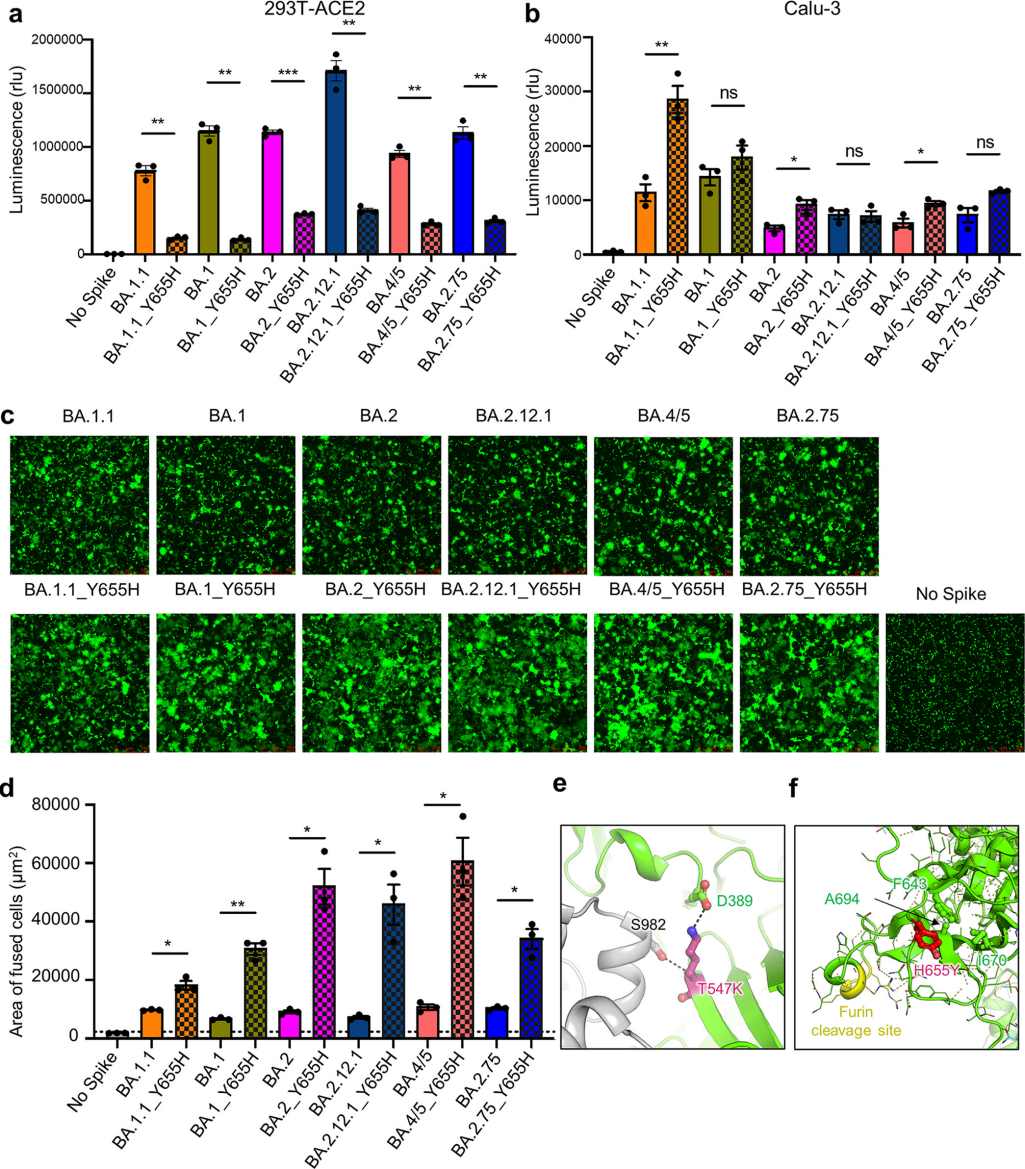
H655Y dictates the endosome entry preference and the low fusogenicity of all major Omicron subvariants. (a) The infectivity of pseudotyped viruses in HEK293T-ACE2 cells is shown (*n* = 3). (b) The infectivity of pseudotyped lentivirus in CaLu-3 cells is also displayed (*n* = 3). (c and d) Syncytium formation assays in HEK293T-ACE2 cells were performed as described in Fig. 3. (c and d) Representative syncytium images (c) and the average area of fused cells (d) are shown (*n* = 3). Scale bars represent 150 μm. (e) Molecular modeling. The T547K mutation introduces a salt-bridge interaction with the D389 located on the base of the RBD and a hydrogen bond with residue S982 in the S2 domain. (f) The BA.1.1 H655Y mutation reduces local conformational flexibility by creating hydrophobic contacts with residues F643, A694, and I670, which may interfere with the furin accessibility to the cleavage site nearby. The bars in panels a, b, and d represent means ± standard error, and significance was determined by two-tailed Student’s *t* test with Welch’s correction. ns, *P* ≥ 0.05; *, *P* < 0.05; **, *P* < 0.01; ***, *P* < 0.001.

The comparable or modestly increased efficiency of furin cleavage in viral producer cells of K547T and Y655H reversion mutants, in contrast to their decreased levels of S1 in the virions, especially Y655H, were indeed surprising, especially given their significantly increased cell-cell fusion activity observed in HEK293T-ACE2 cells. One possibility is that the S1 subunit of these two reversion mutants, especially Y655H, might have increased shedding into culture media during virus production. Indeed, we found that, compared to the parental BA.1.1, K547T, and Y655H exhibited substantially increased levels of S1 shedding into culture media in the presence of treatment by soluble ACE2 (Fig. 3k and l). Of note, the S1 shedding of two other reversion mutants, K679N and H681P, was decreased compared to that of the parental BA.1.1, which appeared to correlate with their relatively low furin cleavage efficiency (Fig. 4k and l). Altogether, these results suggest that the T547K and H655Y mutations in BA.1.1, especially the latter, could stabilize the spike conformation, thus contributing to the decreased fusogenicity and entry efficiency in Calu-3 cells.

### H655Y dictates the endosomal entry and low fusogenicity of other Omicron subvariants.

As in BA.1.1, all previously or currently circulating Omicron subvariants contain the H655Y substitution, but not T547K (Fig. S4). We therefore predicted that the H655Y mutation also governs the entry preference and low fusogenicity of other variants. To this end, we introduced the H655Y reversion mutation into these Omicron subvariants, i.e., BA.1, BA.2, BA.2.12.1, BA.4/5, and BA.2.75, and examined their impact on entry in HEK293T-ACE2 and Calu-3 cells using the same HIV lentiviral pseudotyping system. We observed that the reversion mutation Y655H significantly reduced the entry efficiency of all these Omicron subvariants in HEK293T-ACE2 cells (Fig. 4a) but substantially promoted their entry in Calu-3 cells, although the increase in Calu-3 cells was generally modest or not seen in some cases (Fig. 4b). We also examined the influence of the reversion mutation Y655H on the fusogenicity of these Omicron subvariants using the syncytium formation assay described above. Again, we found that like BA.1.1, Y655H also significantly promoted the S-mediated syncytium formation of BA.1, BA.2, BA.2.12.1, BA.4/5, and BA.2.75 (Fig. 4c and d).

10.1128/mbio.03176-22.4 FIG S4 Schematic representation of Omicron subvariant spike glycoprotein. Only the amino acid substitutions (relative to SARS-CoV-2 D614G S) at the C terminus of S1 and/or near the S1/S2 junction in BA.1.1, BA.1, BA.2, BA.2.12.1, BA.4/5, and BA.2.75 S are indicated. The T547K substitution, which is included only in BA.1.1 and BA.1 S, is highlighted in red. Download FIG S4, TIF file, 2.5 MB. Copyright © 2023 Qu et al. 2023 Qu et al. https://creativecommons.org/licenses/by/4.0/ This content is distributed under the terms of the Creative Commons Attribution 4.0 International license.

### Molecular modeling.

To provide molecular insight into how T547K and H655Y might dictate the low fusogenicity and endosomal entry of the Omicron subvariants, we performed molecular modeling of Omicron BA.1.1 spike protein. Indeed, we found that the T547K mutation could establish a salt-bridge interaction with the residue D389 located on the base of the receptor binding domain (RBD), as well as an interprotomer hydrogen bond with the residue S982 on S2 domain, thus stabilizing its closed conformation and preventing S1 shedding (Fig. 4e). The H655Y mutation located in proximity to the furin cleavage site in S could reduce local structural flexibility by forming hydrophobic interactions with residues F643, I670, and A694 (Fig. 4f), in accordance with the increased shedding of the Y655H reversion mutation (Fig. 3i to l).

## DISCUSSION

Omicron exhibits exceptional immune evasion (1, 7, 33–35) because of the presence of large numbers of mutations in the S protein. We and others have reported that Omicron also exhibits a distinct entry preference, substantially impaired furin cleavage, and decreased cell-cell fusion (23, 25, 36). However, the molecular mechanisms of these distinct features of the Omicron subvariants remain elusive. In this study, we interrogated key mutations that govern the Omicron S-mediated low fusogenicity and endosomal entry. We determined H655Y, and to lesser extent T547K, as the key mutation on the S protein of Omicron that critically governs its preferential endosomal entry route and impaired fusion activity, including for the recently emerged BA.4/5, BA.2.12.1, and BA.2.75 subvariants. Moreover, through molecular modeling, we provide evidence that these mutations likely stabilize the S trimer conformation by forming new molecular interactions. Together, these results provide insights for understanding the altered cellular tropism and pathogenicity for Omicron.

The most important finding of this study is that the altered entry route preference of Omicron is largely determined by the key H655Y mutation. It is well established that SARS-CoV-2 is capable of utilizing either endosomal entry mediated by Cat L/B or plasma membrane entry facilitated by TMPRSS2 (17, 18, 26). However, SARS-CoV-2 entry in primary lung epithelial cells and lung-derived cell lines such as Calu-3 cells is largely TMPRSS2 dependent (18), likely occurring on the plasma membrane. Following its emergence, the Omicron variant BA.1 was shown to have a distinct entry profile, predominantly utilizing the endosomal entry pathway (37), exhibiting poor replication in lower airway-derived primary cells and Calu-3 cells (23), and displaying reduced disease severity (14). We found here that the H655Y mutation governs BA.1.1 entry through endosomes, as suggested by the significant increase in viral infectivity observed in HEK293T-ACE2 cells but substantial reduction in viral infectivity in Calu-3 cells as well as by the increased sensitivity of H655Y bearing variants to E64d, yet with decreased sensitivity to camostat, findings which are also supported by other recent studies (38, 39). However, the impact of H655Y on *in vivo* virus tropism and pathogenicity remains to be investigated. If the altered entry route preference introduced by the H655Y mutation is responsible for the enhanced nasopharynx tropism and reduced pathogenicity of the Omicron subvariants, any reversion of the H655Y mutation in future variants would be of great concern, because such a variant may exhibit enhanced pathogenicity. Careful monitoring of this reversion mutation in the pandemic is warranted.

Membrane fusion is critical for entry of all enveloped viruses. We found that reversion mutations K547T and Y655H significantly promoted BA.1.1 S-mediated cell-cell fusion, whereas forward mutations T547K and H655Y slightly impaired the D614G S-mediated cell-cell fusion, indicating that these two residues critically determine the low fusogenicity of BA.1.1. Quite unexpectedly, T547K does not affect S processing of BA.1.1, and H655Y modestly decreases the furin cleavage efficiency in viral producer cells. Of interest, reversion mutations K547T and Y655H strongly promote S1 shedding into culture media in the presence of soluble ACE2 (sACE2), indicating that T547K and H655Y mutations, especially the latter, likely stabilize the BA.1.1 S trimer conformation. This result correlates with our structural modeling showing that T547K stabilizes the close conformation of S protein, which is consistent with a recent study reporting an extra hydrogen bond between the tyrosine residue at position 655 in S1 and the threonine residue at position 696 in S2 of BA.1 (39). Further structural analyses, including comparisons between K547T/Y655H reversion mutants and the parental BA.1.1 or other Omicron subvariants by cryogenic electron microscopy (cryo-EM) or crystallography, are needed to further elucidate the role of T547K and/or H655Y in Omicron subvariant S conformation.

It is important to note that H655Y mutation has been found to be associated with SARS-CoV-2 infection in cats and minks (20, 40). In addition, the H655Y mutation appears to have arisen independently multiple times in human populations and is a lineage-defining mutation for the Gamma (P.1) SARS-CoV-2 variant in addition to the Omicron subvariants (20). Critically, H655Y is present in all predominant Omicron sublineages, including BA.1.1, BA.1, BA.2, BA.2.12.1, and more recently, BA.4/5 and BA.2.75, indicating that H655Y may improve the viral fitness and the ability to adapt to new hosts, including humans, cats, minks, and others. This notion is further supported by a recent report demonstrating an enhancement of virus infectivity in mice for H655Y-containing viruses (41).

The findings in this work, along with other recent reports, together suggest that the occurrence of mutations at position 655 in S protein of current and future SARS-CoV-2 variants needs to be closely monitored. Additionally, *in vivo* examinations of the impact of the H655Y mutation on virus tropism and pathogenicity are critical and need to be investigated, as any reversion of the H655Y mutation could generate new concern for the course of the COVID-19 pandemic as novel Omicron subvariants continue to emerge.

## MATERIALS AND METHODS

### Cell lines and maintenance.

HEK293T (ATCC CRL-11268, research resource identifier [RRID] CVCL_1926), HEK293T-ACE2 (BEI NR-52511, RRID CVCL_A7UK), and Vero E6 expressing high endogenous ACE2 (Vero-ACE2) (BEI NR-53726, RRID CVCL_A7UJ) supplemented with 1% penicillin/streptomycin (MilliporeSigma, P4333) and 10% (vol/vol) fetal bovine serum (FBS) (Thermo Fisher Scientific, 26140-079) were used. Calu-3 cells, a gift of Estelle Cormet-Boyaka at The Ohio State University, were grown in Eagle’s minimum essential medium (EMEM) (ATCC, 30-2003) supplemented with 1% penicillin/streptomycin and 10% (vol/vol) FBS. HEK293T-ACE2 cells or Vero-ACE2 cells stably expressing TMPRSS2 were generated by transduction of pLX304 vectors expressing TMPRSS2 (a gift from Siyuan Ding at the Washington University in St. Louis, MO) and selection by blasticidine S hydrochloride (MilliporeSigma, 15205) (10 μg/mL for HEK293T-ACE2 cells and 7.5 μg/mL for Vero-ACE2 cells) for 7 to 14 days. All cell lines in this study were maintained at 37°C in the presence of 5% CO_2_.

### Plasmids and constructs.

Constructs harboring different mutations were generated by long PCR mutagenesis based on pcDNA3.1-SARS-CoV-2-Flag-S-Flag-D614G, pcDNA3.1-SARS-CoV-2-Flag-S-Flag-BA.1.1, pcDNA3.1-SARS-CoV-2-Flag-S-Flag-BA.1, pcDNA3.1-SARS-CoV-2-Flag-S-Flag-BA.2, pcDNA3.1-SARS-CoV-2-Flag-S-Flag-BA.2.12.1, pcDNA3.1-SARS-CoV-2-Flag-S-Flag-BA.4/5, or pcDNA3.1-SARS-CoV-2-Flag-S-Flag-BA.2.75 (13). The HIV-1 NL4.3-inGluc was from Marc Johnson at the University of Missouri (Columbia, MO, USA).

### Generation of BA.1 FL-mNG and related mutant viruses.

Infectious cDNA clones of USA-WA1/2020 and Omicron (BA.1) SARS-CoV-2 were generated using PCR-based mutagenesis as previously described (29, 30). To construct spike mutant K547T or Y655H mutant viruses, nucleotide substitutions were introduced through a standard mutagenesis approach into subclone pcc1-CoV-2-BA.1-F567 containing the spike gene of SARS-CoV-2 BA.1. The full-length infectious cDNA clone of SARS-CoV-2 was assembled by *in vitro* ligation of three contiguous cDNA fragments following the previously described protocol (22, 30). *In vitro* transcription was performed to synthesize full-length viral genomic RNA. The RNA transcripts were electroporated in Vero E6 cells expressing TMPRSS2 (purchased from SEKISUI XenoTech, LLC) to recover the mutant viruses. Viruses were rescued 2 to 4 days after electroporation and served as P0 stock. P0 stock was further passaged once on Vero E6 cells expressing TMPRSS2 to produce P1 stock. The spike gene was sequenced from all P1 stock viruses to validate the mutations. P1 stock was titrated by plaque assay on Vero E6 cells expressing TMPRSS2 and used for subsequent experiments. All virus preparation was carried out at a biosafety level 3 (BSL-3) facility at the University of Texas Medical Branch at Galveston, TX.

### Virus production and infection.

HEK293T cells were transfected with 0.7 μg of different Spike plasmids along with 1.4 μg of HIV-1-NL4.3-inGluc at a 1:2 ratio using polyethylenimine transfection (42). Then, 48 to 72 h posttransfection, the culture supernatants were harvested. After removing the cell debris by spinning down at 3,000 × *g* for 10 min, the viruses were aliquoted and stored at −80°C.

Pseudovirions were transduced into various cell lines; 6 h postransduction, the media were changed. Gaussia luciferase activity was measured 48 to 96 h after infection to determine the relative infectivity or entry efficiency of the indicated viruses.

For the inhibition assay using pseudotyped viruses, HEK293T-ACE2-TMPRSS2 cells were pretreated with the indicated concentrations of EST/E-64D (E64d) (Sigma, 330005) or camostat mesylate (camostat) (Sigma, SML0057) for 1 h, followed by transduction with pseudovirions of interest in the presence of the same concentrations of the drugs. After changing the media at 6 h postinfection (hpi), the luciferase activity was measured at 48 and 72 hpi.

For the inhibition assay using infectious viruses, Vero-ACE2-TMPRSS2 cells were pretreated with the indicated concentrations of EST/E-64D (E64d) (Sigma, 330005) or camostat mesylate (camostat) (Sigma, SML0057) for 1 h, followed by infection with infectious viruses in the presence of the same concentrations of the inhibitors for 24 h. Subsequently, the cells were fixed with 3.7% formaldehyde for 1 h and analyzed by a Life Technologies Attune NxT flow cytometer.

### Flow cytometry.

HEK293T cells used for the production of virions were washed with 1× phosphate-buffered saline (PBS) once, detached with 5 mM EDTA in PBS, and washed with washing buffer (1× PBS containing 2% FBS) twice, followed by fixing with 3.7% formaldehyde for 10 min. Then the cells were incubated with anti-SARS-CoV-2 spike antibodies (T62) for 1 h on ice. After three washes with cold washing buffer, the cells were incubated with fluorescein isothiocyanate (FITC)-conjugated anti-rabbit IgG antibodies for 1 h. Subsequently, the cells were washed twice and analyzed with a Life Technologies Attune NxT flow cytometer.

### Syncytium formation assay.

HEK293T-ACE2 cells were cotransfected with GFP constructs and parental D614G S, Omicron S, or their related mutants, followed by imaging the syncytium formation under a Leica DMi8 fluorescence microscope after 24 h of transfection. The cell-cell fusion efficiency was analyzed by measuring areas of the fused cells from three representative images using Leica Application Suit X software. Scale bars represent 150 μm.

### Western blotting.

Western blotting was conducted as previously described (28). Briefly, cells were collected and lysed in 200 μL of RIPA buffer (50 mM Tris [pH 7.5], 150 mM NaCl, 1 mM EDTA, Nonidet P-40, 0.1% SDS) in the presence of protease inhibitor cocktail (MilliporeSigma, P8340), followed by clarification at 13,200 rpm for 10 min and boiling for 10 min at 100°C with 1× SDS loading buffer. To determine the spike content in virion particles, pseudovirus supernatant was collected, filtered, and purified by ultracentrifugation through a 20% sucrose cushion. The purified virions were dissolved in 1× SDS loading buffer. Subsequently, the samples were separated by 10% SDS-PAGE gels, transferred to polyvinylidene difluoride (PVDF) membranes, and immunoblotted with anti-S1 (Sino Biological, 40150-T62), anti-S2 (Sino Biological, 40590-T62), anti-GAPDH (Santa Cruz, sc-47724), and anti-HIV-1 p24 (anti-p24 [NIH ARP-1513]) antibodies, followed by immunoblotting with anti-mouse-IgG-peroxidase (Sigma, A5278) or anti-rabbit-IgG-horseradish peroxidase (HRP) (Sigma, A9169) antibodies.

### Soluble ACE2-induced S1 shedding assay.

HEK293T cells were transfected with the indicated Omicron spike constructs and were treated or untreated with soluble ACE2-Fc (sACE2-Fc) at 37°C for 4 h. Subsequently, the cell culture media and cells were collected, and anti-FLAG beads (Sigma, F2426) were used to precipitate S1 subunit in the media. Following immune-precipitation, the samples were separated on 10% SDS-PAGE gel and probed with anti-S1 (Sino Biological, 40150-T62), anti-S2 (Sino Biological, 40590-T62), and anti-GAPDH (Santa Cruz, sc-47724). Anti-mouse-IgG-peroxidase (Sigma, A5278) and anti-rabbit-IgG-HRP (Sigma, A9169) were used as secondary antibodies.

### Structural modeling and analysis.

Homology modeling of Omicron spike protein was performed on the SWISS-MODEL server with the cryo-EM structure of SARS-CoV2 G614 strain spike (PDB 7KRR) as the template. The resulting homo-trimer spike structure has one RBD in the up conformation and the other two RBDs in the down conformation. Residue examination and renumbering were carried out manually with the program Coot. Interprotomer interaction analysis was performed with the PDBePISA server. Molecular contacts of Omicron mutants were examined and illustrated with the program PyMOL.

### Statistical analysis.

All experiments were conducted in at least three independent replications except for those specifically indicated. Statistical significance analyses were performed in GraphPad Prism 9 (San Diego, CA) and are referenced in the figure legends and include one-way analysis of variance (ANOVA) with Bonferroni’s posttests to compute statistical significance between multiple groups for multiple comparisons (Fig. 1b to g, Fig. 3b, c, f, g, i, j, and l, Fig. S3b and c, Fig. S4b, c, e, and f), and an unpaired, two-tailed Student’s *t* test with Welch’s correction was used (Fig. 4a, b, and d).

### Data availability.

The data reported in this paper will be shared by the corresponding author upon request. Any additional information required to reanalyze the data reported in this paper is available from the corresponding author upon request.

## References

[1] Viana R, Moyo S, Amoako DG, Tegally H, Scheepers C, Althaus CL, Anyaneji UJ, Bester PA, Boni MF, Chand M, Choga WT, Colquhoun R, Davids M, Deforche K, Doolabh D, Du Plessis L, Engelbrecht S, Everatt J, Giandhari J, Giovanetti M, Hardie D, Hill V, Hsiao NY, Iranzadeh A, Ismail A, Joseph C, Joseph R, Koopile L, Kosakovsky Pond SL, Kraemer MUG, Kuate-Lere L, Laguda-Akingba O, Lesetedi-Mafoko O, Lessells RJ, Lockman S, Lucaci AG, Maharaj A, Mahlangu B, Maponga T, Mahlakwane K, Makatini Z, Marais G, Maruapula D, Masupu K, Matshaba M, Mayaphi S, Mbhele N, Mbulawa MB, Mendes A, Mlisana K, et al. 2022. Rapid epidemic expansion of the SARS-CoV-2 Omicron variant in southern Africa. Nature 603:679–686. doi:10.1038/d41586-021-03832-5. 35042229 PMC8942855

[2] Yu J, Collier AY, Rowe M, Mardas F, Ventura JD, Wan H, Miller J, Powers O, Chung B, Siamatu M, Hachmann NP, Surve N, Nampanya F, Chandrashekar A, Barouch DH. 2022. Neutralization of the SARS-CoV-2 Omicron BA.1 and BA.2 Variants. N Engl J Med 386:1579–1580. doi:10.1056/NEJMc2201849. 35294809 PMC9006770

[3] Liu L, Iketani S, Guo Y, Chan JF, Wang M, Liu L, Luo Y, Chu H, Huang Y, Nair MS, Yu J, Chik KK, Yuen TT, Yoon C, To KK, Chen H, Yin MT, Sobieszczyk ME, Huang Y, Wang HH, Sheng Z, Yuen KY, Ho DD. 2022. Striking antibody evasion manifested by the Omicron variant of SARS-CoV-2. Nature 602:676–681. doi:10.1038/s41586-021-04388-0. 35016198

[4] Evans JP, Zeng C, Qu P, Faraone J, Zheng YM, Carlin C, Bednash JS, Zhou T, Lozanski G, Mallampalli R, Saif LJ, Oltz EM, Mohler PJ, Xu K, Gumina RJ, Liu SL. 2022. Neutralization of SARS-CoV-2 Omicron sub-lineages BA.1, BA.1.1, and BA.2. Cell Host Microbe 30:1093–1102.e3. doi:10.1016/j.chom.2022.04.014. 35526534 PMC9035359

[5] Carreño JM, Alshammary H, Tcheou J, Singh G, Raskin AJ, Kawabata H, Sominsky LA, Clark JJ, Adelsberg DC, Bielak DA, Gonzalez-Reiche AS, Dambrauskas N, Vigdorovich V, Alburquerque B, Amoako AA, Banu R, Beach KF, Bermúdez-González MC, Cai GY, Ceglia I, Cognigni C, Farrugia K, Gleason CR, van de Guchte A, Kleiner G, Khalil Z, Lyttle N, Mendez WA, Mulder LCF, Oostenink A, Rooker A, Salimbangon AT, Saksena M, Paniz-Mondolfi AE, Polanco J, Srivastava K, Sather DN, Sordillo EM, Bajic G, van Bakel H, Simon V, Krammer F, PSP-PARIS Study Group. 2022. Activity of convalescent and vaccine serum against SARS-CoV-2 Omicron. Nature 602:682–688. doi:10.1038/d41586-021-03846-z. 35016197

[6] Perez-Then E, Lucas C, Monteiro VS, Miric M, Brache V, Cochon L, Vogels CBF, Malik AA, De la Cruz E, Jorge A, De Los Santos M, Leon P, Breban MI, Billig K, Yildirim I, Pearson C, Downing R, Gagnon E, Muyombwe A, Razeq J, Campbell M, Ko AI, Omer SB, Grubaugh ND, Vermund SH, Iwasaki A. 2022. Neutralizing antibodies against the SARS-CoV-2 Delta and Omicron variants following heterologous CoronaVac plus BNT162b2 booster vaccination. Nat Med 28:481–485. doi:10.1038/s41591-022-01705-6. 35051990 PMC8938264

[7] Planas D, Saunders N, Maes P, Guivel-Benhassine F, Planchais C, Buchrieser J, Bolland WH, Porrot F, Staropoli I, Lemoine F, Pere H, Veyer D, Puech J, Rodary J, Baele G, Dellicour S, Raymenants J, Gorissen S, Geenen C, Vanmechelen B, Wawina-Bokalanga T, Marti-Carreras J, Cuypers L, Seve A, Hocqueloux L, Prazuck T, Rey FA, Simon-Loriere E, Bruel T, Mouquet H, Andre E, Schwartz O. 2022. Considerable escape of SARS-CoV-2 Omicron to antibody neutralization. Nature 602:671–675. doi:10.1038/s41586-021-04389-z. 35016199

[8] Xia H, Zou J, Kurhade C, Cai H, Yang Q, Cutler M, Cooper D, Muik A, Jansen KU, Xie X, Swanson KA, Shi PY. 2022. Neutralization and durability of 2 or 3 doses of the BNT162b2 vaccine against Omicron SARS-CoV-2. Cell Host Microbe 30:485–488.e3. doi:10.1016/j.chom.2022.02.015. 35245438 PMC8853806

[9] Stegger M, Edslev SM, Sieber RN, Ingham AC, Ng KL, Tang MHE, Alexandersen S, Fonager J, Legarth R, Utko M, Wilkowski B, Gunalan V, Bennedbæk M, Byberg-Grauholm J, Møller CH, Christiansen LE, Svarrer CW, Ellegaard K, Baig S, Ohannesen TB, Espenhain L, Skov R, Cohen AS, Larsen NB, Sørensen KM, White ED, Lillebaek T, Ullum H, Krause TG, Fomsgaard A, Ethelberg S, Rasmussen M. 2022. Occurrence and significance of Omicron BA.1 infection followed by BA.2 reinfection. medRxiv. doi:10.1101/2022.02.19.22271112.

[10] Cao Y, Yisimayi A, Jian F, Song W, Xiao T, Wang L, Du S, Wang J, Li Q, Chen X, Yu Y, Wang P, Zhang Z, Liu P, An R, Hao X, Wang Y, Wang J, Feng R, Sun H, Zhao L, Zhang W, Zhao D, Zheng J, Yu L, Li C, Zhang N, Wang R, Niu X, Yang S, Song X, Chai Y, Hu Y, Shi Y, Zheng L, Li Z, Gu Q, Shao F, Huang W, Jin R, Shen Z, Wang Y, Wang X, Xiao J, Xie XS. 2022. BA.2.12.1, BA.4 and BA.5 escape antibodies elicited by Omicron infection. Nature 608:593–602. doi:10.1038/s41586-022-04980-y. 35714668 PMC9385493

[11] Hachmann NP, Miller J, Collier AY, Ventura JD, Yu J, Rowe M, Bondzie EA, Powers O, Surve N, Hall K, Barouch DH. 2022. Neutralization escape by SARS-CoV-2 Omicron subvariants BA.2.12.1, BA.4, and BA.5. N Engl J Med 387:86–88. doi:10.1056/NEJMc2206576. 35731894 PMC9258748

[12] Wang Q, Guo Y, Iketani S, Nair MS, Li Z, Mohri H, Wang M, Yu J, Bowen AD, Chang JY, Shah JG, Nguyen N, Chen Z, Meyers K, Yin MT, Sobieszczyk ME, Sheng Z, Huang Y, Liu L, Ho DD. 2022. Antibody evasion by SARS-CoV-2 Omicron subvariants BA.2.12.1, BA.4 and BA.5. Nature 608:603–608. doi:10.1038/s41586-022-05053-w. 35790190 PMC9385487

[13] Qu P, Faraone J, Evans JP, Zou X, Zheng YM, Carlin C, Bednash JS, Lozanski G, Mallampalli RK, Saif LJ, Oltz EM, Mohler PJ, Gumina RJ, Liu SL. 2022. Neutralization of the SARS-CoV-2 Omicron BA.4/5 and BA.2.12.1 subvariants. N Engl J Med 386:2526–2528. doi:10.1056/NEJMc2206725. 35704428 PMC9258774

[14] Halfmann PJ, Iida S, Iwatsuki-Horimoto K, Maemura T, Kiso M, Scheaffer SM, Darling TL, Joshi A, Loeber S, Singh G, Foster SL, Ying B, Case JB, Chong Z, Whitener B, Moliva J, Floyd K, Ujie M, Nakajima N, Ito M, Wright R, Uraki R, Warang P, Gagne M, Li R, Sakai-Tagawa Y, Liu Y, Larson D, Osorio JE, Hernandez-Ortiz JP, Henry AR, Ciuoderis K, Florek KR, Patel M, Odle A, Wong LR, Bateman AC, Wang Z, Edara VV, Chong Z, Franks J, Jeevan T, Fabrizio T, DeBeauchamp J, Kercher L, Seiler P, Gonzalez-Reiche AS, Sordillo EM, Chang LA, van Bakel H, Consortium Mount Sinai Pathogen Surveillance (PSP) study group., et al. 2022. SARS-CoV-2 Omicron virus causes attenuated disease in mice and hamsters. Nature 603:687–692. doi:10.1038/s41586-022-04441-6. 35062015 PMC8942849

[15] McMahan K, Giffin V, Tostanoski LH, Chung B, Siamatu M, Suthar MS, Halfmann P, Kawaoka Y, Piedra-Mora C, Jain N, Ducat S, Kar S, Andersen H, Lewis MG, Martinot AJ, Barouch DH. 2022. Reduced pathogenicity of the SARS-CoV-2 omicron variant in hamsters. Med (N Y) 3:262–268.e4. doi:10.1016/j.medj.2022.03.004. 35313451 PMC8926874

[16] Zhou P, Yang XL, Wang XG, Hu B, Zhang L, Zhang W, Si HR, Zhu Y, Li B, Huang CL, Chen HD, Chen J, Luo Y, Guo H, Jiang RD, Liu MQ, Chen Y, Shen XR, Wang X, Zheng XS, Zhao K, Chen QJ, Deng F, Liu LL, Yan B, Zhan FX, Wang YY, Xiao GF, Shi ZL. 2020. A pneumonia outbreak associated with a new coronavirus of probable bat origin. Nature 579:270–273. doi:10.1038/s41586-020-2012-7. 32015507 PMC7095418

[17] Hoffmann M, Kleine-Weber H, Schroeder S, Kruger N, Herrler T, Erichsen S, Schiergens TS, Herrler G, Wu NH, Nitsche A, Muller MA, Drosten C, Pöhlmann S. 2020. SARS-CoV-2 cell entry depends on ACE2 and TMPRSS2 and is blocked by a clinically proven protease inhibitor. Cell 181:271–280.e8. doi:10.1016/j.cell.2020.02.052. 32142651 PMC7102627

[18] Peacock TP, Goldhill DH, Zhou J, Baillon L, Frise R, Swann OC, Kugathasan R, Penn R, Brown JC, Sanchez-David RY, Braga L, Williamson MK, Hassard JA, Staller E, Hanley B, Osborn M, Giacca M, Davidson AD, Matthews DA, Barclay WS. 2021. The furin cleavage site in the SARS-CoV-2 spike protein is required for transmission in ferrets. Nat Microbiol 6:899–909. doi:10.1038/s41564-021-00908-w. 33907312 PMC7619196

[19] Mykytyn AZ, Breugem TI, Riesebosch S, Schipper D, van den Doel PB, Rottier RJ, Lamers MM, Haagmans BL. 2021. SARS-CoV-2 entry into human airway organoids is serine protease-mediated and facilitated by the multibasic cleavage site. Elife 10:e64508. doi:10.7554/eLife.64508. 33393462 PMC7806259

[20] Escalera A, Gonzalez-Reiche AS, Aslam S, Mena I, Laporte M, Pearl RL, Fossati A, Rathnasinghe R, Alshammary H, van de Guchte A, Farrugia K, Qin Y, Bouhaddou M, Kehrer T, Zuliani-Alvarez L, Meekins DA, Balaraman V, McDowell C, Richt JA, Bajic G, Sordillo EM, Dejosez M, Zwaka TP, Krogan NJ, Simon V, Albrecht RA, van Bakel H, Garcia-Sastre A, Aydillo T. 2022. Mutations in SARS-CoV-2 variants of concern link to increased spike cleavage and virus transmission. Cell Host Microbe 30:373–387.e7. doi:10.1016/j.chom.2022.01.006. 35150638 PMC8776496

[21] Saito A, Irie T, Suzuki R, Maemura T, Nasser H, Uriu K, Kosugi Y, Shirakawa K, Sadamasu K, Kimura I, Ito J, Wu J, Iwatsuki-Horimoto K, Ito M, Yamayoshi S, Loeber S, Tsuda M, Wang L, Ozono S, Butlertanaka EP, Tanaka YL, Shimizu R, Shimizu K, Yoshimatsu K, Kawabata R, Sakaguchi T, Tokunaga K, Yoshida I, Asakura H, Nagashima M, Kazuma Y, Nomura R, Horisawa Y, Yoshimura K, Takaori-Kondo A, Imai M, Genotype t, Phenotype Japan C, Tanaka S, Nakagawa S, Ikeda T, Fukuhara T, Kawaoka Y, Sato K, Genotype to Phenotype Japan (G2P-Japan) Consortium. 2022. Enhanced fusogenicity and pathogenicity of SARS-CoV-2 Delta P681R mutation. Nature 602:300–306. doi:10.1038/s41586-021-04266-9. 34823256 PMC8828475

[22] Liu Y, Liu J, Johnson BA, Xia H, Ku Z, Schindewolf C, Widen SG, An Z, Weaver SC, Menachery VD, Xie X, Shi PY. 2022. Delta spike P681R mutation enhances SARS-CoV-2 fitness over Alpha variant. Cell Rep 39:110829. doi:10.1016/j.celrep.2022.110829. 35550680 PMC9050581

[23] Meng B, Abdullahi A, Ferreira IATM, Goonawardane N, Saito A, Kimura I, Yamasoba D, Gerber PP, Fatihi S, Rathore S, Zepeda SK, Papa G, Kemp SA, Ikeda T, Toyoda M, Tan TS, Kuramochi J, Mitsunaga S, Ueno T, Shirakawa K, Takaori-Kondo A, Brevini T, Mallery DL, Charles OJ, Baker S, Dougan G, Hess C, Kingston N, Lehner PJ, Lyons PA, Matheson NJ, Ouwehand WH, Saunders C, Summers C, Thaventhiran JED, Toshner M, Weekes MP, Maxwell P, Shaw A, Bucke A, Calder J, Canna L, Domingo J, Elmer A, Fuller S, Harris J, Hewitt S, Kennet J, Jose S, Kourampa J, The CITIID-NIHR BioResource COVID-19 Collaboration, et al. 2022. Altered TMPRSS2 usage by SARS-CoV-2 Omicron impacts infectivity and fusogenicity. Nature 603:706–714. doi:10.1038/s41586-022-04474-x. 35104837 PMC8942856

[24] Du X, Tang H, Gao L, Wu Z, Meng F, Yan R, Qiao S, An J, Wang C, Qin FX. 2022. Omicron adopts a different strategy from Delta and other variants to adapt to host. Signal Transduct Target Ther 7:45. doi:10.1038/s41392-022-00903-5. 35145066 PMC8830988

[25] Qu P, Faraone JN, Evans JP, Zou X, Zheng Y-M, Carlin C, Bednash JS, Lozanski G, Mallampalli RK, Saif LJ, Oltz EM, Mohler PJ, Gumina RJ, Liu S-L. 2022. Differential Evasion of Delta and Omicron immunity and enhanced fusogenicity of SARS-CoV-2 Omicron BA.4/5 and BA.2.12.1 subvariants. BioRxiv doi:10.1101/2022.05.16.492158.

[26] Bestle D, Heindl MR, Limburg H, Van Lam van T, Pilgram O, Moulton H, Stein DA, Hardes K, Eickmann M, Dolnik O, Rohde C, Klenk HD, Garten W, Steinmetzer T, Böttcher-Friebertshäuser E. 2020. TMPRSS2 and furin are both essential for proteolytic activation of SARS-CoV-2 in human airway cells. Life Sci Alliance 3:e202000786. doi:10.26508/lsa.202000786. 32703818 PMC7383062

[27] Gobeil SM, Janowska K, McDowell S, Mansouri K, Parks R, Manne K, Stalls V, Kopp MF, Henderson R, Edwards RJ, Haynes BF, Acharya P. 2021. D614G mutation alters SARS-CoV-2 spike conformation and enhances protease cleavage at the S1/S2 junction. Cell Rep 34:108630. doi:10.1016/j.celrep.2020.108630. 33417835 PMC7762703

[28] Zeng C, Evans JP, Pearson R, Qu P, Zheng YM, Robinson RT, Hall-Stoodley L, Yount J, Pannu S, Mallampalli RK, Saif L, Oltz E, Lozanski G, Liu SL. 2020. Neutralizing antibody against SARS-CoV-2 spike in COVID-19 patients, health care workers, and convalescent plasma donors. JCI Insight 5:e143213. doi:10.1172/jci.insight.143213. 33035201 PMC7710271

[29] Pitts J, Li J, Perry JK, Du Pont V, Riola N, Rodriguez L, Lu X, Kurhade C, Xie X, Camus G, Manhas S, Martin R, Shi PY, Cihlar T, Porter DP, Mo H, Maiorova E, Bilello JP. 2022. Remdesivir and GS-441524 retain antiviral activity against Delta, Omicron, and other emergent SARS-CoV-2 variants. Antimicrob Agents Chemother 66:e0022222. doi:10.1128/aac.00222-22. 35532238 PMC9211395

[30] Xie X, Muruato A, Lokugamage KG, Narayanan K, Zhang X, Zou J, Liu J, Schindewolf C, Bopp NE, Aguilar PV, Plante KS, Weaver SC, Makino S, LeDuc JW, Menachery VD, Shi PY. 2020. An infectious cDNA clone of SARS-CoV-2. Cell Host Microbe 27:841–848.e3. doi:10.1016/j.chom.2020.04.004. 32289263 PMC7153529

[31] Rajah MM, Hubert M, Bishop E, Saunders N, Robinot R, Grzelak L, Planas D, Dufloo J, Gellenoncourt S, Bongers A, Zivaljic M, Planchais C, Guivel-Benhassine F, Porrot F, Mouquet H, Chakrabarti LA, Buchrieser J, Schwartz O. 2021. SARS-CoV-2 Alpha, Beta, and Delta variants display enhanced spike-mediated syncytia formation. EMBO J 40:e108944. doi:10.15252/embj.2021108944. 34601723 PMC8646911

[32] Johnson BA, Xie X, Bailey AL, Kalveram B, Lokugamage KG, Muruato A, Zou J, Zhang X, Juelich T, Smith JK, Zhang L, Bopp N, Schindewolf C, Vu M, Vanderheiden A, Winkler ES, Swetnam D, Plante JA, Aguilar P, Plante KS, Popov V, Lee B, Weaver SC, Suthar MS, Routh AL, Ren P, Ku Z, An Z, Debbink K, Diamond MS, Shi PY, Freiberg AN, Menachery VD. 2021. Loss of furin cleavage site attenuates SARS-CoV-2 pathogenesis. Nature 591:293–299. doi:10.1038/s41586-021-03237-4. 33494095 PMC8175039

[33] Garcia-Beltran WF, St Denis KJ, Hoelzemer A, Lam EC, Nitido AD, Sheehan ML, Berrios C, Ofoman O, Chang CC, Hauser BM, Feldman J, Roederer AL, Gregory DJ, Poznansky MC, Schmidt AG, Iafrate AJ, Naranbhai V, Balazs AB. 2022. mRNA-based COVID-19 vaccine boosters induce neutralizing immunity against SARS-CoV-2 Omicron variant. Cell 185:457–466.e4. doi:10.1016/j.cell.2021.12.033. 34995482 PMC8733787

[34] Kuhlmann C, Mayer CK, Claassen M, Maponga T, Burgers WA, Keeton R, Riou C, Sutherland AD, Suliman T, Shaw ML, Preiser W. 2022. Breakthrough infections with SARS-CoV-2 omicron despite mRNA vaccine booster dose. Lancet 399:625–626. doi:10.1016/S0140-6736(22)00090-3. 35063123 PMC8765759

[35] Pulliam JRC, van Schalkwyk C, Govender N, von Gottberg A, Cohen C, Groome MJ, Dushoff J, Mlisana K, Moultrie H. 2022. Increased risk of SARS-CoV-2 reinfection associated with emergence of Omicron in South Africa. Science 376:6593. doi:10.1126/science.abn4947. PMC8995029 35289632

[36] Zeng C, Evans JP, Qu P, Faraone J, Zheng YM, Carlin C, Bednash JS, Zhou T, Lozanski G, Mallampalli R, Saif LJ, Oltz EM, Mohler P, Xu K, Gumina RJ, Liu SL. 2021. Neutralization and Stability of SARS-CoV-2 Omicron variant. bioRxiv. doi:10.1101/2021.12.16.472934.

[37] Pia L, Rowland-Jones S. 2022. Omicron entry route. Nat Rev Immunol 22:144. doi:10.1038/s41577-022-00681-9. PMC8790940 35082449

[38] Hu B, Chan JF, Liu H, Liu Y, Chai Y, Shi J, Shuai H, Hou Y, Huang X, Yuen TT, Yoon C, Zhu T, Zhang J, Li W, Zhang AJ, Zhou J, Yuan S, Zhang BZ, Yuen KY, Chu H. 2022. Spike mutations contributing to the altered entry preference of SARS-CoV-2 Omicron BA.1 and BA.2. Emerg Microbes Infect 11:2275–2287. doi:10.1080/22221751.2022.2117098. 36039901 PMC9542985

[39] Yamamoto M, Tomita K, Hirayama Y, Inoue JI, Kawaguchi Y, Gohda J. 2022. SARS-CoV-2 Omicron spike H655Y mutation is responsible for enhancement of the endosomal entry pathway and reduction of cell surface entry pathways. BioRxiv doi:10.1101/2022.03.21.485084.

[40] Braun KM, Moreno GK, Halfmann PJ, Hodcroft EB, Baker DA, Boehm EC, Weiler AM, Haj AK, Hatta M, Chiba S, Maemura T, Kawaoka Y, Koelle K, O’Connor DH, Friedrich TC. 2021. Transmission of SARS-CoV-2 in domestic cats imposes a narrow bottleneck. PLoS Pathog 17:e1009373. doi:10.1371/journal.ppat.1009373. 33635912 PMC7946358

[41] Zhu Y, Zhou W, Niu Z, Sun J, Zhang Z, Li Q, Zheng Y, Wang C, Gao L, Sun Q. 2022. Long-range enhancement of N501Y-endowed mouse infectivity of SARS-CoV-2 by the non-RBD mutations of Ins215KLRS and H655Y. Biol Direct 17:14. doi:10.1186/s13062-022-00325-x. 35658928 PMC9167559

[42] Cui Z, Liu P, Wang N, Wang L, Fan K, Zhu Q, Wang K, Chen R, Feng R, Jia Z, Yang M, Xu G, Zhu B, Fu W, Chu T, Feng L, Wang Y, Pei X, Yang P, Xie XS, Cao L, Cao Y, Wang X. 2022. Structural and functional characterizations of infectivity and immune evasion of SARS-CoV-2 Omicron. Cell 185:860–871.e13. doi:10.1016/j.cell.2022.01.019. 35120603 PMC8786603

[43] Mlcochova P, Kemp SA, Dhar MS, Papa G, Meng B, Ferreira IATM, Datir R, Collier DA, Albecka A, Singh S, Pandey R, Brown J, Zhou J, Goonawardane N, Mishra S, Whittaker C, Mellan T, Marwal R, Datta M, Sengupta S, Ponnusamy K, Radhakrishnan VS, Abdullahi A, Charles O, Chattopadhyay P, Devi P, Caputo D, Peacock T, Wattal C, Goel N, Satwik A, Vaishya R, Agarwal M, Mavousian A, Lee JH, Bassi J, Silacci-Fegni C, Saliba C, Pinto D, Irie T, Yoshida I, Hamilton WL, Sato K, Bhatt S, Flaxman S, James LC, Corti D, Piccoli L, Barclay WS, Rakshit P, CITIID-NIHR BioResource COVID-19 Collaboration, et al. 2021. SARS-CoV-2 B.1.617.2 Delta variant replication and immune evasion. Nature 599:114–119. doi:10.1038/s41586-021-03944-y. 34488225 PMC8566220

