# Supplementary figures and images for: Determinants and Mechanisms of the Low Fusogenicity and High Dependence on Endosomal Entry of Omicron Subvariants
Source: mBio. 2023 Jan 10;14(1):e03176-22. doi: 10.1128/mbio.03176-22 (PMC9972997; doi:10.1128/mbio.03176-22)

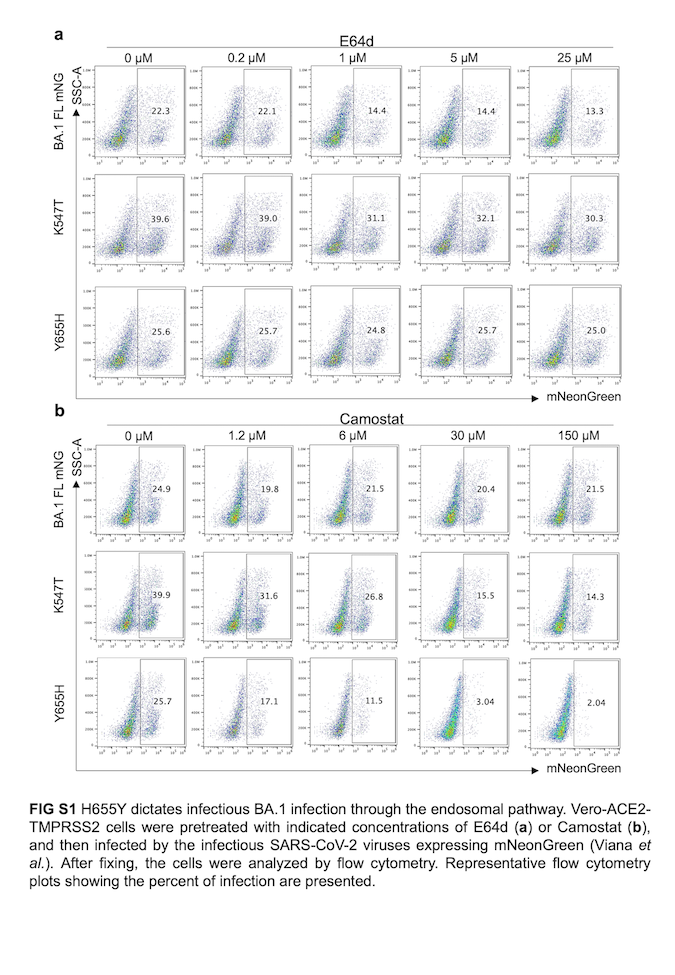

Supplement: FIG S1 [file mbio.03176-22-s0001.tif]

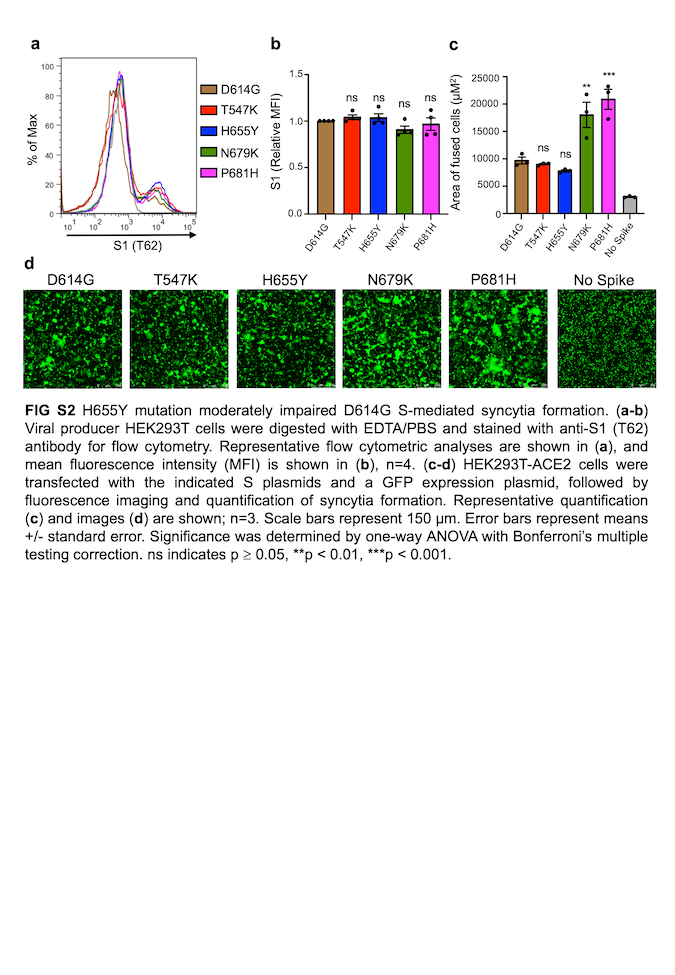

Supplement: FIG S2 [file mbio.03176-22-s0002.tif]

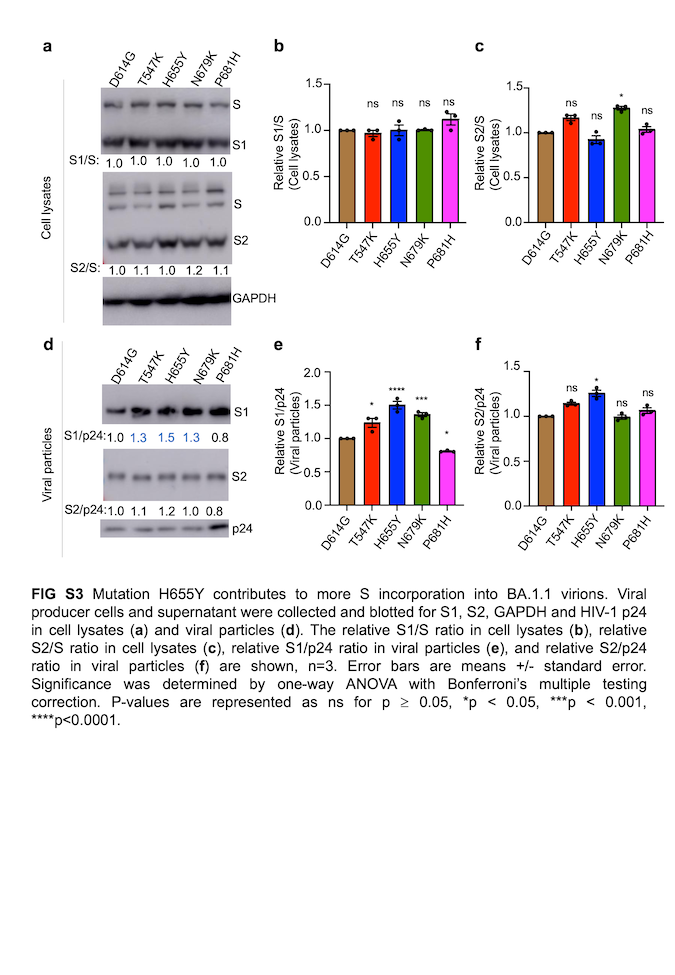

Supplement: FIG S3 [file mbio.03176-22-s0003.tif]

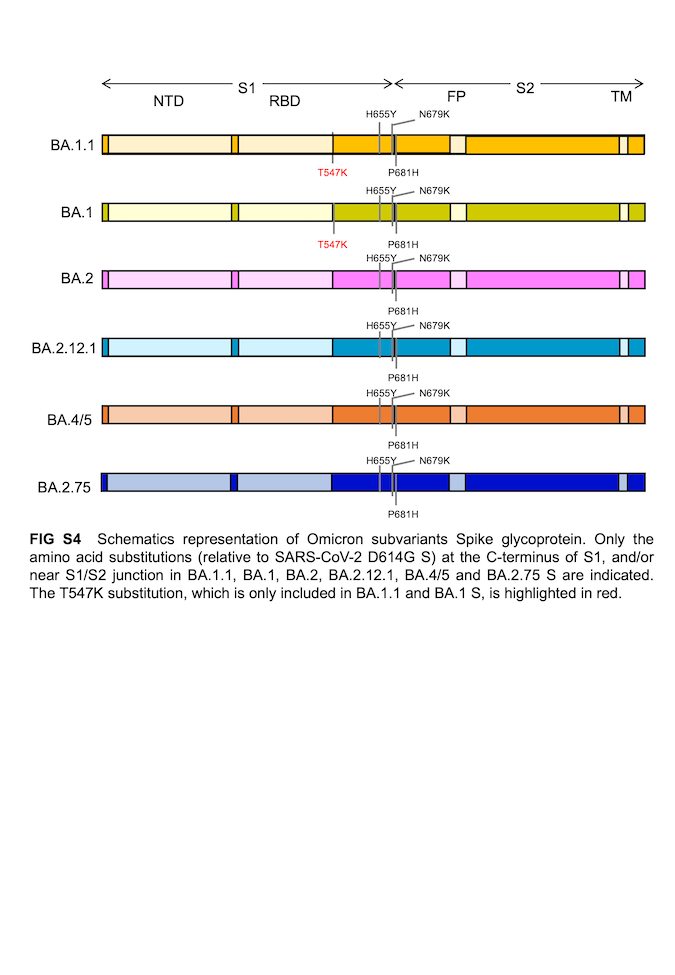

Supplement: FIG S4 [file mbio.03176-22-s0004.tif]
